# RRAM-Based Neuromorphic Devices for Artificial-Intelligence Hardware: Device Physics, Materials, Processing, and Packaging

**DOI:** 10.3390/mi17091032

**Published:** 2026-08-29

**Authors:** Sung Gyu Pyo

**Affiliations:** School of Integrative Engineering, Chung-Ang University, 84 Heukseok-ro, Dongjak-gu, Seoul 06974, Republic of Korea; sgpyo@cau.ac.kr

**Keywords:** RRAM, resistive switching, neuromorphic computing, memristor, synaptic device, in-memory computing

## Abstract

Resistive random-access memory (RRAM) has emerged as one of the most promising device platforms for neuromorphic, in-memory computing because its two-terminal metal–insulator–metal (MIM) structure can reproduce the weight-update behavior of biological synapses while remaining compatible with mainstream CMOS processing. This review summarizes the current state of RRAM-based neuromorphic technology from four complementary perspectives: device physics, materials, fabrication processes, and packaging. We first describe the operating principles of filamentary and interface-type RRAM, including the forming/set/reset switching sequence, and the two dominant analytical frameworks used to describe the reset transition—the ion-migration model and the thermally driven filament-dissolution model. We then review the switching-layer and electrode materials that have been most widely investigated such as HfO_x_, TiO_x_, TaO_x_, ZnO, ZrO_2_, and Cu/Ag-based conductive-bridge systems, together with representative bilayer and doped architectures reported for synaptic devices. The biological functions that RRAM can emulate are discussed alongside the non-ideal characteristics that currently limit on-chip training accuracy, with emphasis on separating device-to-device from cycle-to-cycle variability and on the workload-dependent nature of endurance and retention requirements. We further summarize the process technologies used to integrate RRAM into large-scale, CMOS-compatible arrays, including atomic layer deposition, interfacial oxygen-reservoir engineering, low-thermal-budget back-end-of-line integration, and three-dimensional vertical RRAM patterning, and discuss the advanced packaging strategies such as 2.5D/3D heterogeneous integration, chiplet architectures, thermal-interface materials, and nanostructured underfills required to manage the power density and interconnect demands of large synaptic arrays, distinguishing solutions that have been demonstrated specifically for RRAM neuromorphic arrays from those that remain general advanced-packaging concepts. Finally, RRAM is benchmarked against competing emerging non-volatile memories, and the key research directions such as three-terminal memtransistor architectures, three-dimensional integration with high-performance selectors, and hardware–algorithm co-design that will determine whether RRAM-based neuromorphic hardware can move from laboratory demonstrations to on-device AI, autonomous systems, and large-scale artificial-neural-network accelerators are outlined. Relative to prior reviews that focus primarily on RRAM device physics or on switching-layer materials in isolation, the distinctive contribution of this review is an explicit, cross-layer synthesis that connects device-level non-idealities to their consequences for wafer-scale process integration and for advanced 2.5D/3D packaging—a combination that, to our knowledge, has not been jointly treated in the recent review literature on RRAM-based neuromorphic hardware.

## 1. Introduction

The rapid expansion of artificial intelligence (AI), big data, the Internet of Things, smart factories, and autonomous mobility has driven an explosive increase in the volume of data that semiconductor systems must store and process. Because conventional charge-storage-based memories are approaching their physical scaling limits, extensive research has been devoted to non-charge-based, next-generation non-volatile memories such as ferroelectric RAM (FeRAM), magnetic RAM (MRAM), phase-change RAM (PCRAM), and resistive RAM (RRAM). Among these, RRAM is particularly attractive because of its simple fabrication, its two-terminal metal–insulator–metal (MIM) structure, excellent scalability, nanosecond-scale switching speed, long data retention, and compatibility with existing CMOS process flows [1,2,3,4,5,6].

At the same time, conventional computing systems remain built around the von Neumann architecture, in which the processing unit (CPU/GPU) and the memory unit are physically separated. As deep-learning models have grown explosively in size, the resulting data traffic between memory and processor—the so-called von Neumann bottleneck—together with the associated power consumption, has become a critical bottleneck for further performance scaling. Neuromorphic computing, which mimics the structure and information-processing strategy of the biological brain, has therefore attracted increasing attention as a next-generation computing paradigm. Neuromorphic systems aim to realize processing-in-memory (PIM), in which synapse- and neuron-like elements are connected in parallel so that computation and memory occur within the same physical location. In a crossbar-array implementation, Kirchhoff’s current law and Ohm’s law can be exploited directly so that a vector–matrix multiplication (VMM) operation,

$$I_{out}=\sum_{j}V_{j}\cdot G_{ij}$$

is carried out in parallel with O(1) time complexity with respect to the number of multiply–accumulate operations performed by the array itself. This O(1) scaling applies specifically to the analog dot-product step; the end-to-end latency of a practical crossbar operation also includes DAC/ADC conversion time, wire and column-multiplexing delay, RC settling time, and, for high-precision inputs or weights, multiple bit-sliced read/write cycles and peripheral-circuit overhead, none of which scale as O(1). We use the O(1) description here only for the core analog computation and return to these peripheral-circuit costs and to system-level energy metrics in Section 5 and Section 8. RRAM is one of the key enabling devices for this type of in-memory computing: its simple two-terminal structure and its reliable, voltage-controlled resistive-switching (memristive) behavior make it well suited to emulate the weight-modulation function of a biological synapse

The biological motivation for this approach is direct. The human brain consists of roughly 10^11^ neurons interconnected by approximately 10^15^ synapses and performs highly parallel, energy-efficient computation and pattern recognition using an information-processing scheme entirely different from that of a von Neumann machine. Neurons integrate the inputs from many synapses and generate a corresponding output spike, while synapses store and adapt information by changing the strength of the connection in response to a sequence of neural activity. This behavior is directly analogous to the resistance-switching characteristic of a memristive device, in which stored data change as a function of the applied electrical stimulus. From the standpoint of AI algorithms, RRAM-based neuromorphic hardware is relevant to more than one generation of neural-network models. Mainstream deep neural networks and Transformer-based models—regarded as a second generation of artificial neural network—operate on continuous, floating-point activations, whereas spiking neural networks (SNNs), often described as a third generation of artificial intelligence, instead generate a discrete “spike” only when a neuron’s membrane potential exceeds a threshold and are otherwise silent. Because an RRAM cell only needs to update its conductance when it actually receives such a spike, an SNN built from RRAM synapses can, in principle, leave idle cells in a static, non-switching state between events, which is the basis for the often-cited claim that RRAM is “naturally suited” to sparse, event-driven computation. This claim should be qualified in three respects, however: (i) the static conductance state still draws a small read/leakage current whenever the row/column lines are biased for inference, so standby power approaches, but does not exactly reach, zero; (ii) peripheral CMOS circuitry (neuron integrate-and-fire circuits, spike encoders/decoders, and any DAC/ADC stages) continues to consume power independent of spike sparsity and can dominate total energy at low spike rates; and (iii) the sparsity advantage is realized only if the surrounding digital control logic is itself event-driven (e.g., asynchronous, address-event-representation readout) rather than being polled on a fixed clock, which is not automatic in every reported RRAM-SNN system. We revisit these overheads quantitatively in the energy-efficiency discussion of Section 8. The RRAM conductance can further evolve in an analog fashion as a function of the time interval, Δt, between pre- and post-synaptic spikes, reproducing spike-timing-dependent plasticity directly at the device level; this allows unsupervised, biologically inspired learning rules to be implemented in hardware without an explicit backpropagation algorithm. In addition, RRAM crossbar arrays are being actively investigated as analog multiply–accumulate engines for accelerating large, already-trained AI models, and as a platform for AI quantization research.

A further motivation is that conventional artificial neural networks suffer from catastrophic forgetting, in which learning new information erases previously learned knowledge. The biological brain avoids this problem by forming new synaptic connections while largely preserving existing ones. If the short-term plasticity (STP) and long-term plasticity (LTP) behaviors of RRAM can be jointly engineered and combined with appropriate learning algorithms, important weights can be consolidated while unimportant weights remain flexible—providing a physical substrate for lifelong learning systems that continuously adapt to a changing environment. Viewed from this perspective, RRAM-based neuromorphic computing is progressing beyond simply adapting von-Neumann-oriented algorithms to hardware (“hardware-aware AI”) and moving toward architectures in which algorithm and hardware are unified in a brain-inspired manner.

Despite this promise, RRAM devices still exhibit a range of non-ideal characteristics—nonlinear and asymmetric conductance updates, pronounced device-to-device and cycle-to-cycle variability, sneak-path leakage current in crossbar arrays, IR-drop at large array scale, and limited endurance and retention—that must be addressed before RRAM-based neuromorphic hardware can be deployed at scale. This review therefore examines the current status of RRAM-based neuromorphic devices from four perspectives: (i) device operating principles and switching models; (ii) switching-layer materials, electrodes, and representative synaptic-device case studies; (iii) the non-ideal characteristics that limit on-chip learning accuracy; and (iv) the process and packaging technologies required to integrate RRAM arrays into manufacturable, large-scale neuromorphic hardware. The review concludes by comparing RRAM with competing emerging non-volatile memories and by outlining the key research directions for future RRAM-based neuromorphic systems. This review is complementary to, rather than duplicative of, several recent reviews on RRAM-based and light-driven neuromorphic hardware. Bousoulas et al. [7] recently reviewed emerging light-driven (optoelectronic) neuromorphic hardware, with a device-physics focus on photonic and opto-memristive switching mechanisms; Ielmini and Pedretti [8] reviewed the application requirements and system-level specifications of RRAM for memory and computing; and Shooshtari et al. [9] reviewed memristors for in-memory computing and spiking neural networks from an algorithm-and-circuit perspective. Xia et al. [10] separately reviewed low-power memristor materials and applications for neuromorphic computing more broadly.The present review differs from each of these in scope: rather than treating device physics, materials, on-chip computing, or packaging as separate literature, it follows a single class of device (oxide-based RRAM) across the full stack from filament-level switching physics, through materials and electrode engineering, to wafer-level process integration and 2.5D/3D packaging, explicitly cross-referencing how a limitation identified at one level (e.g., filament-level Joule heating in the “Material–Process Co-Optimization” discussion of Section 6) reappears as a constraint at another (chip-level thermal management in the “Thermal Management for Densely Integrated RRAM” discussion of Section 7). This cross-layer framing, rather than any single section in isolation, is the main added value relative to the reviews cited above.

## 2. RRAM Device Structure and Operating Principles

**Metal–Insulator–Metal Structure and Switching Modes.**

An RRAM cell consists of a top electrode (TE) and a bottom electrode (BE) separated by an insulating switching layer, forming a metal–insulator–metal (MIM) stack. The insulating layer can be an organic compound or, more commonly, one of a wide range of binary or multinary metal oxides or chalcogenide-based materials, while the electrodes are typically noble metals, electrically conductive non-metals, or metal nitrides.

Two broad device architectures are used to realize this switching behavior: **Filamentary RRAM**, in which a localized conducting path is formed by the aggregation of oxygen vacancies or by the migration of metal ions (e.g., Ag, Cu). Filamentary devices can support multi-level-cell (MLC) operation and typically switch quickly. **Non-filamentary (interface-type) RRAM**, in which the resistance change occurs uniformly across the device area as a result of interfacial redox reactions or changes in the Schottky-barrier height. Because the conductance changes continuously over the whole active area rather than through abrupt filament rupture, interface-type devices tend to show superior analog linearity.

**Figure 1:**
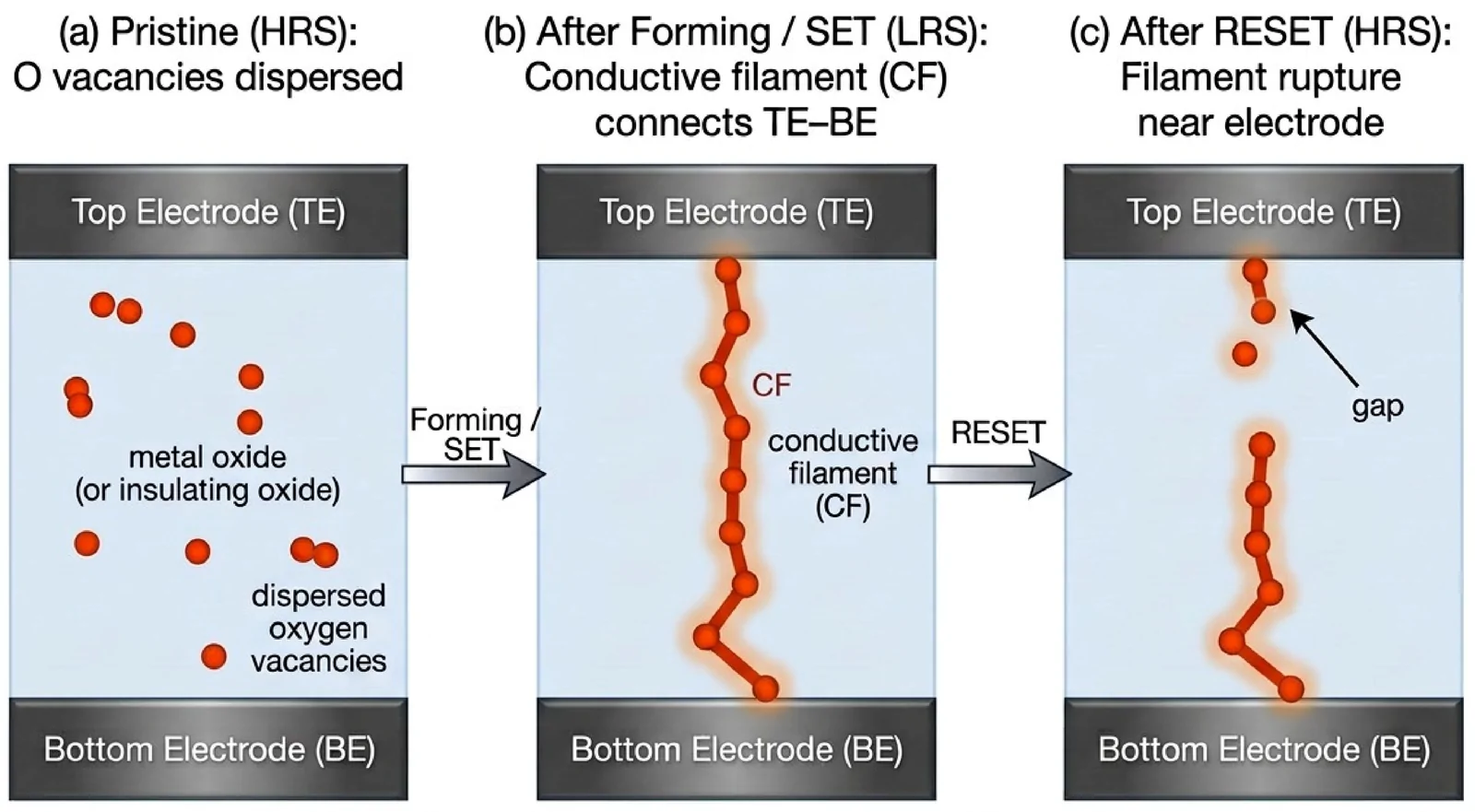
Schematic of the RRAM MIM stack and its principal switching states, as described in the “Metal–Insulator–Metal Structure and Switching Modes” part of Section 2. Schematic of the RRAM metal–insulator–metal (MIM) stack and its three principal switching states: (**a**) the pristine, high-resistance state (HRS) with dispersed oxygen vacancies; (**b**) the low-resistance state (LRS) after forming/SET, in which a conductive filament (CF) bridges the top and bottom electrodes; and (**c**) the HRS restored after RESET, in which the filament ruptures near one electrode [1,2,3,4,5,6].

RRAM operation is generally based on the formation or dissolution of a conductive filament (CF) within the insulating layer. A pristine device starts in a high-resistance state (HRS). Applying a voltage larger than the eventual operating voltage—the forming voltage, V_f—is usually required once, in a step called electroforming, to establish the first conductive filament and enable subsequent, lower-voltage switching cycles. After forming, applying a SET voltage (V_set) switches the device from the HRS to a low-resistance state (LRS), while applying a RESET voltage (V_reset) switches the device back from the LRS to the HRS; the LRS and HRS correspond to the ON and OFF states of the device, respectively (Figure 1). Depending on whether the polarity of the applied voltage matters, RRAM switching is classified as unipolar (switching direction depends only on the magnitude of the applied voltage) or bipolar (switching direction depends on the polarity of the applied voltage). These two switching modes are governed by different physical mechanisms at the electrode/oxide interface: the thermal-dissolution model largely explains unipolar switching, while the ion-migration model largely explains bipolar switching. This unipolar–thermal/bipolar–ionic pairing describes which mechanism tends to dominate and is not a statement that the two mechanisms are mutually exclusive. Oxygen-ion migration is itself thermally activated (see the “Switching Models” discussion in Section 2), so any ionic-drift process generates Joule heat that can locally assist filament rupture, and conversely, a thermally dissolving filament changes the local resistance and, therefore, the local field and ionic drift rate; the two switching models describe which of these coupled electrochemical, thermal, and field-driven processes is rate-limiting under a given bias/polarity condition, not two independent physical channels. We adopt this framing throughout the “Switching Models” discussion in Section 2.

Physically, the forming/set transition can be understood as a dielectric soft breakdown of the insulating layer. Under a high electric field (>10 MV/cm), oxygen ions are displaced from the oxide lattice and drift toward the anode interface, leaving oxygen vacancies within the bulk oxide. The resulting local oxygen deficiency leads to the formation of a conductive filament composed of oxygen vacancies or, in some material systems, precipitated metal. The displaced oxygen ions are discharged at the electrode/oxide interface—which acts as an oxygen-ion reservoir—by reacting with a noble or oxidizable anode material. In the LRS, current flows through this conductive filament. During RESET, oxygen ions instead recombine with the oxygen vacancies (or oxidize the metallic precipitate) and migrate back into the bulk oxide, returning the device to the HRS. Repeated SET/RESET cycling repeatedly connects and disconnects the filament; because the compliance current is usually limited (by a compliance circuit or a series transistor/resistor during forming), the filament diameter can be controlled and hard breakdown of the insulating layer can be avoided.

The growth kinetics of the conductive filament can be described by a rate equation for the effective filament diameter, Φ:

$$\frac{d\Phi }{dt}\propto Aexp\left(-\frac{E_{A}}{k_{B}T}\right)$$

where A is an Arrhenius pre-factor, E_A is the activation energy for ion migration, and T is the local filament temperature. This relation indicates that the filament growth rate is limited by the supply of migrating cations and is ultimately controlled by the ion-hopping rate, which itself depends on temperature through the given activation-energy barrier.

**Switching Models.**

**Ion-migration model.** For both unipolar and bipolar metal-oxide RRAM, the SET process begins with dielectric soft breakdown, during which oxygen deficiency in the bulk oxide leads to filament formation via oxygen vacancies or metal precipitates. The displaced O^2−^ ions accumulate at the electrode interface, forming an oxygen reservoir; during the subsequent RESET process, these O^2−^ ions are supplied back to annihilate oxygen vacancies or to oxidize metallic precipitates. In bipolar RESET, drift and diffusion act cooperatively, so that O^2−^ ions move from the anode into the bulk oxide and straightforwardly rupture the filament; in unipolar RESET, by contrast, these two driving forces oppose one another. The corresponding O^2−^ ion flux, J_D (cm^−2^ s^−1^), can be expressed through a diffusion coefficient derived from the one-dimensional rigid-point-ion model of Mott and Gurney, from which the ion drift velocity and the time-dependent O^2−^ concentration can, in turn, be obtained. In this framework, the relaxation time associated with the recombination of O^2−^ ions with oxygen vacancies (or with the oxidation of metallic precipitates) is typically taken to be on the order of 10 ns in the specific HfO_2_-based devices and bias conditions originally modeled by Ielmini and co-workers [11,12,13,14]; this value is not a universal constant. It depends on the activation energy for oxygen-ion migration and on the local filament temperature, both of which are material- and stack-dependent, so measured relaxation/switching times for other oxide systems, film thicknesses, or applied-field conditions can differ from this figure by orders of magnitude. We retain the 10 ns figure here only as an order-of-magnitude illustration of the model, not as a representative value for RRAM switching layers in general.

**Figure 2:**
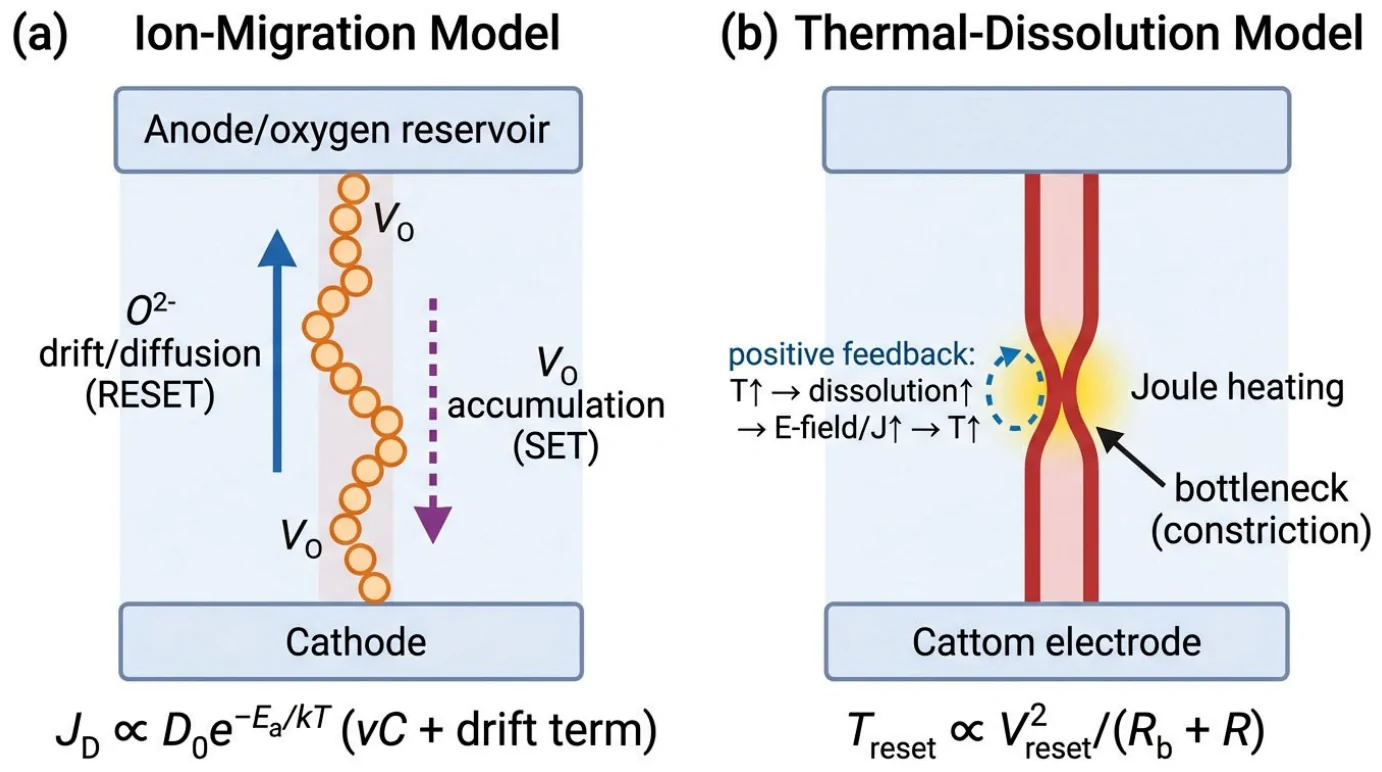
Schematic comparison of the ion-migration and thermal-dissolution switching models. Schematic comparison of the two switching models discussed in the “Switching Models” part of Section 2: (**a**) the ion-migration model, in which O^2−^ ions drift/diffuse between the bulk oxide and the electrode oxygen reservoir, and (**b**) the thermal-dissolution model, in which localized Joule heating at the filament bottleneck drives a positive-feedback constriction process [11,12,13,14,15,16].

**Thermal-dissolution model.** To explain the universal RESET behavior of unipolar RRAM, a model based on thermally activated dissolution of the conductive filament is required. In this model, the temperature within the filament rises during a voltage sweep or pulse, increasing the probability that excess metal species will oxidize and thereby locally reducing conductivity. Russo [15,16] et al. proposed a thermal-dissolution model for the RESET transition of unipolar, metal-oxide-based RRAM, showing that the abrupt resistance change observed during RESET is accelerated by positive feedback between local Joule heating and thermal dissolution of the filament at its narrowest constriction (bottleneck). The local temperature required to trigger RESET at the filament, T_reset, and the corresponding RESET voltage, V_reset—defined as the voltage needed to raise the local filament temperature to this critical value—can be obtained from the steady-state solution of the Fourier heat-conduction equation. As Joule heating increases along the I–V curve, thermally driven dissolution causes the filament to constrict; this locally enhances the electric field and current density, further increasing Joule dissipation and temperature and thereby accelerating dissolution. This positive-feedback loop underlies the abrupt current drop observed during RESET (Figure 2).

## 3. RRAM Materials and Electrode Engineering

**Figure 3:**
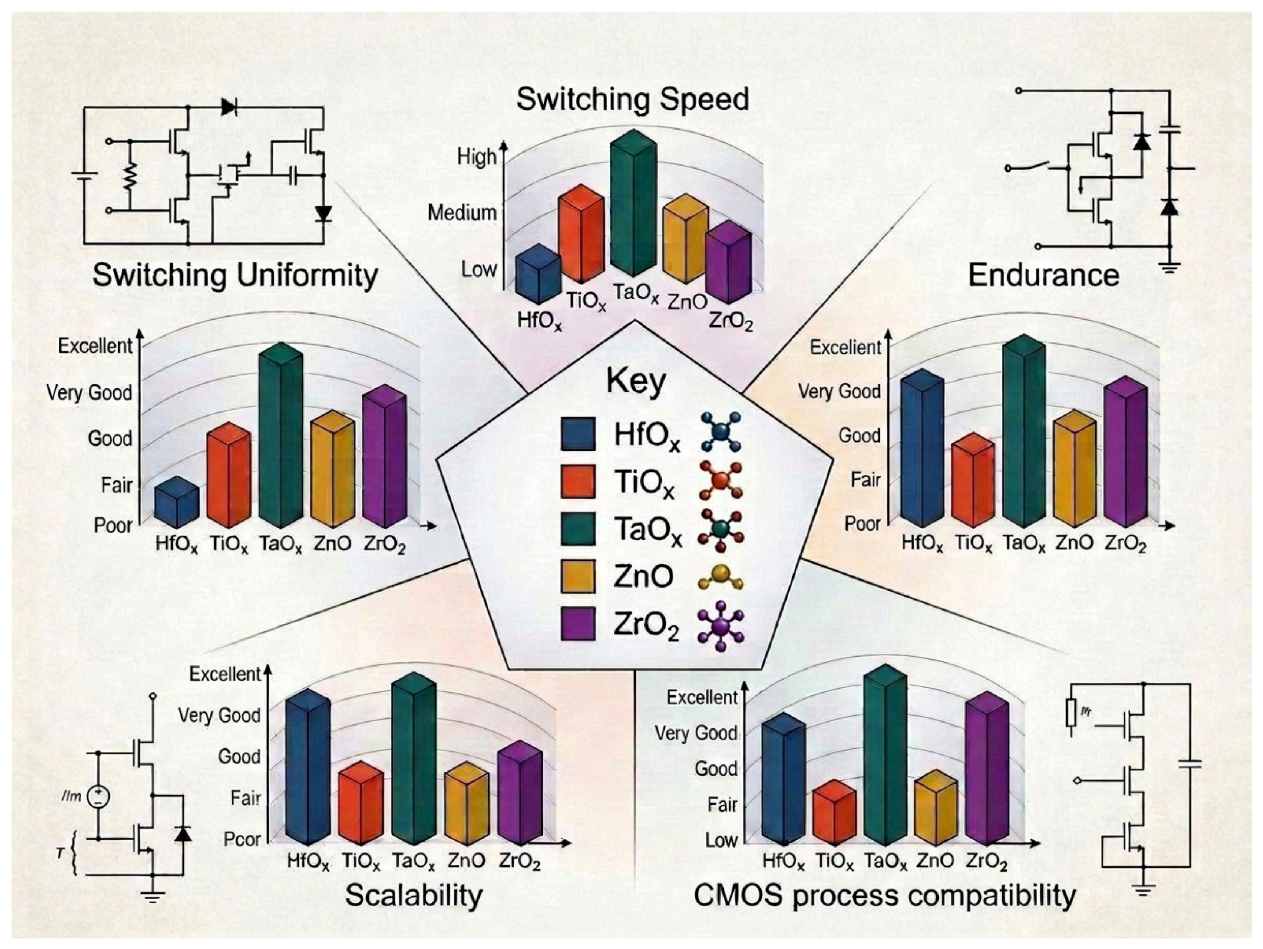
Qualitative comparison of RRAM switching-layer oxide materials discussed in Section 3. Qualitative 3. (HfO_x_, TiO_x_, TaO_x_, ZnO, ZrO_2_) across five figures of merit: switching speed, endurance, CMOS process compatibility, scalability, and switching uniformity. Scores are a qualitative synthesis of the trends discussed in the text rather than a benchmarked, quantitative dataset [17,18,19,20,21,22,23,24,25,26,27,28,29,30,31,32,33,34,35,36,37,38,39,40,41,42,43,44].

RRAM switching-layer materials can be broadly divided into organic and inorganic classes. Organic switching layers still face unresolved reliability issues—including high SET/RESET voltages, elevated power consumption, and broad voltage-distribution spread—so inorganic oxide- and chalcogenide-based materials, which offer simpler processing, more stable performance, and lower cost, dominate current research and are the focus of this section (Figure 3).

**Oxide-Based Switching Layers (OxRAM).**

Oxide-based memory does not require high-temperature annealing or deposition and is therefore widely regarded as the most suitable insulator choice for RRAM devices.

HfO_2_ combines a high dielectric constant with high density, good ductility, and corrosion resistance, and has long been studied as a replacement for SiO_2_. It is most commonly deposited by thermal atomic layer deposition (ALD), electron-beam evaporation, or sputtering; ALD films in particular are essentially pinhole-free, dense, and low in contamination. Because the metal–organic precursors used in ALD decompose fully only at elevated temperature, HfO_2_ is conventionally deposited near 200 °C, motivating substantial research into lower-temperature deposition routes. Kim [17] et al. showed that HfO_2_ films deposited at a low temperature (80 °C) by O_2_-plasma-assisted PEALD exhibit lower contamination, higher density, and lower leakage current than films deposited by thermal ALD. Su [18] et al. demonstrated that the forming voltage of HfO_2_-based RRAM can be effectively reduced either by lengthening the rise time of the forming voltage waveform or by increasing the forming temperature; they found that a short accumulation time (SAT) occurs before forming at elevated temperature, whereas a longer accumulation time (LAT) at lower temperature is associated with a lower HRS resistance. Chen [19] et al. and Zhao [20] et al. separately reported multi-level-cell (MLC) switching in W/Zr/HfO_2_/TiN and TiN/HfO_2_/Pt stacks, respectively; the latter study also introduced a multi-step forming technique that suppresses forming-current overshoot and enables low-voltage, low-current switching, improving the relative standard deviation of the resistance level of a 3-bit-per-cell HfO_2_ device by up to 80% compared with single-pulse programming. Feng [21] et al. fabricated a vertical, low-power TiN/Ti/HfO_2_/TiN ReRAM stack in which a limited number of oxygen ions near the Ti/HfO_2_ interface govern resistive switching in the low-power operating mode. Long [22] et al. analyzed the statistics of the RESET voltage and RESET current of unipolar Pt/HfO_2_/Pt devices, reporting that their statistical distributions are governed by the initial resistance distribution. More recently, Shooshtari et al. demonstrated spike-timing-dependent plasticity and multi-timescale synaptic consolidation directly in HfO_2_-based memristors, showing that a single HfO_x_ device can reproduce both the fast, reversible weight change associated with short-term plasticity and a slower, pulse-history-dependent consolidation step analogous to long-term memory formation [23].

TiO_2_ is attractive as an RRAM switching layer because of its high charge-storage capacity over long operating times and its fast resistive switching. Compared with other high-permittivity oxides such as HfO_2_, ZrO_2_, NiO, and ZnO, TiO_2_ offers a relatively high capacitance and storage capacity at scaled dimensions and a wide bandgap (3.2 eV for the anatase phase and 3.0 eV for rutile), which lowers both the current level and the required compliance current relative to narrower-bandgap dielectrics. The relatively high resistivity of TiO_2_, however, still limits its electron-transport properties. Yildiz [24] et al. found that TiO_2_ deposited with water vapor as the reactive gas shows electron-transport properties 10^8^–10^9^ times higher than films deposited using oxygen, consistent with increasing carrier density at higher oxygen-vacancy concentration; dense TiO_2_ films typically also require annealing above 200 °C. Amorphous TiO_2_ (a-TiO_2_) films deposited by plasma-enhanced ALD (PEALD), however, can achieve excellent thickness and roughness control, allowing lower processing temperatures and improved device reliability [25,26,27]. Tsigkourakos [28] et al. and Bousoulas [29] et al. reported multi-level-cell switching in TiO_2_−x-based RRAM: embedding Pt nanocrystals within the oxide film enables ultra-low-power operation in the nanowatt range at operating currents below 200 nA, because the embedded Pt nanocrystals locally enhance the probability of oxygen-vacancy formation, improving switching variability and enabling low-power applications through local field enhancement; a relatively thin filament with high oxygen-vacancy density was found to be central to enabling multi-level operation. Yoshida [30] et al. fabricated and investigated bipolar-switching Pt/rutile-TiO_2_/TiN devices for resistive-memory applications; the same device stack was also shown to support unipolar switching, with the bipolar-mode current typically 10–200 times lower than the unipolar-mode current, giving bipolar switching an advantage in terms of low operating current. Bature et al. subsequently combined modeling with experimental verification of TiO_2_-based RRAM switching and used the same device to demonstrate spike-timing-dependent plasticity, providing a more direct link between the TiO_2_ switching characteristics discussed above and neuromorphic device operation than the earlier memory-oriented studies [31].

TaO_x_ offers excellent endurance and low power consumption in resistive switching. Its properties depend strongly on crystal structure and oxygen composition, and pulsed-laser deposition (PLD) is a preferred technique because the Ta/O ratio and crystallinity can be controlled through the oxygen partial pressure and deposition temperature in the growth chamber. [32,33] Lee [34] et al. demonstrated an asymmetric, passive-switching TaO_x_-based device using an intrinsic Schottky barrier to suppress leakage current, achieving good scalability and low RESET current; a plasma-oxidized bilayer structure was shown to localize the switching region, yielding excellent endurance and switching speed. In this device, a TaO_2_−x layer was deposited by reactive sputtering of a Ta metal target in an oxygen/argon gas mixture, followed by formation of a Ta_2_O_5_−x layer using oxygen plasma, with the plasma process enabling precise control of the Ta_2_O_5_−x layer thickness and sheet resistance. An intrinsic Schottky barrier exists between the top Pt electrode and the Ta_2_O_5_−x layer in the insulating state, while an ohmic contact forms in the LRS, giving a symmetric current profile in the LRS but an asymmetric profile in the HRS—an advantage for high-density applications. Prakash [35] et al. theoretically investigated multi-level resistance variability in nanoscale, MLC-mode TaO_x_-based RRAM, attributing the lower nonlinearity observed at low compliance current to trap-assisted conduction associated with a low oxygen-vacancy concentration; the resistance variability depends both on filament size and on the oxygen-vacancy concentration within the filament, as supported by Monte Carlo calculations of the LRS distribution at various compliance currents, and suggesting that a denser filament (in terms of oxygen vacancies) can improve LRS variability even at a low compliance current. Lee [36] et al. fabricated and characterized a high-reliability, MLC TaO_x_-based RRAM device using a triple-layer stack (base layer/oxygen-exchange layer/barrier layer) targeting storage-class-memory applications. A switchable-mode TaO_x_ device has also been reported, which can be operated either in a bipolar memristive mode or in a complementary-resistive-switch (CRS) mode within the same cell, using the distinct threshold voltages of the two modes to combine non-destructive reading (bipolar mode) with sneak-path-suppressing programming (CRS mode) in a single-device solution to the array-level reliability issues discussed in Section 5 [37].

ZnO is attractive because of its high dielectric constant (k ≈ 10) [38,39] and fab-friendly processing. Among the many candidate metal oxides, ZnO offers low cost, a wide bandgap, low synthesis temperature, controllable electrical behavior, stable electrochemical activity, biocompatibility, and environmental friendliness. It can be grown in a wide variety of one-dimensional morphologies (nanowires, nanoribbons/belts, hierarchical, tubular, nanosheet, nanopropeller, nanohelix, and nanoring structures), which, together with its favorable properties, make it attractive for piezoelectric transducers, biosensors, chemical and gas sensors, photodetectors, optoelectronics, surface-acoustic-wave devices, and transparent conductive oxides. Han [40] et al. fabricated an Au/ZnO/ITO RRAM device on a glass substrate to investigate transparent-electronics applications; because ZnO is a wide-bandgap (3.37 eV) n-type semiconductor, it is transparent in the visible range, and ZnO-based RRAM has consequently been actively studied for transparent electronic devices. A more recent ZnO-based device has also been reported to exhibit self-rectifying behavior through an added ITO semiconductor-like layer, in which an energy-band-diagram-based rectification mechanism is used to suppress sneak-path current without a separate selector element, and synaptic learning behaviors relevant to neuromorphic computing were demonstrated on the same device [41].

ZrO_2_ has been investigated for gate-dielectric applications because of its high dielectric constant, simple composition, and ease of fabrication, although the device yield of non-stoichiometric zirconium oxide is generally low; a variety of approaches—use of stoichiometric ZrO_2_, embedding nanocrystals, Cu-impurity doping, and Zr+ ion or Au implantation—have been proposed to improve device yield. Wang [42] et al. reported reproducible bipolar resistive switching (RS) in Ti/ZrO_2_/Pt devices and observed that the forming voltage depends on the ZrO_2_ thickness, with thicker ZrO_2_ sustaining a higher breakdown voltage; representative bipolar RS behavior was also demonstrated under various forming-current compliance conditions. Lei [43] et al. reported bipolar RRAM switching characteristics in Ti/ZrO_2_/Pt devices, investigating the switching and conduction mechanisms separately. Chen [44] et al. proposed an algorithm to widen the I_set/I_reset ratio and operating margin of ZrO_2_-based OxRAM; while conventional approaches achieve a data resolution of the I_set/I_reset ratio spanning only two to three orders of magnitude within a 4 V (±2 V) operating margin, the proposed algorithm achieves a data resolution spanning three to five orders of magnitude within an 8 V operating margin.

**Electrode Engineering.**

The electrode contributes to RRAM switching through several distinct physical functions that are not always separated explicitly in the device literature: (i) oxygen scavenging/reservoir behavior, in which the electrode reversibly stores and releases oxygen ions exchanged with the switching layer during SET/RESET; (ii) redox activity, in which the electrode metal itself oxidizes or reduces (e.g., Cu, Ag active electrodes) and participates directly in filament formation; (iii) work-function control, which sets the Schottky-barrier height at the electrode/oxide interface and therefore the interface-limited leakage and rectification behavior; (iv) Schottky-barrier modulation by an interfacial reaction layer (e.g., a Ti-induced TiO_x_ interlayer) that is distinct from the bulk work function of the electrode metal; and (v) active-metal supply for conductive-bridge (ECM) devices, where the electrode is consumed to form the filament rather than merely templating it. The examples below are grouped by material family for continuity with the “Oxide-Based Switching Layers (OxRAM)” discussion in Section 3, but each is annotated with which of these functions it primarily illustrates.

RRAM devices consist of a top and a bottom electrode. The bottom electrode influences device behavior through its work function, electronegativity, oxygen affinity, and interfacial reaction and interdiffusion with the switching layer; noble metals such as Pt, Au, and Ru, as well as Ti, TiN, and TaN, are commonly used (function iii, work-function control). TiN electrodes are widely used as effective oxygen reservoirs (function i) because they can control the distribution of mobile oxygen ions and oxygen vacancies within the oxide; the chemical structure of a TiN electrode comprises nitride, titanium-dioxide, and oxynitride components, and its oxygen-storage capacity depends mainly on the nitride and oxynitride content, so different TiN deposition processes give rise to different oxygen-storage capacities and different resistive-switching behaviors. Sun [45] et al. reported that a crystallized (200)-oriented TiN electrode, achieved through epitaxial growth on a CrRu intermediate layer, provides better oxygen-storage capacity than amorphous TiN.

The use of a copper top electrode on a TiO_2_–Pt stack has been shown to give bipolar switching through the formation of a localized conductive filament (function ii, redox/active-metal supply); Yang [46] et al. concluded that copper diffusion and filament formation are the dominant switching mechanisms in Cu/TiO_2_/Pt devices. A programmable-metallization-cell (PMC) memory device using an oxidized Cu–Ti alloy bottom electrode achieved an ON/OFF ratio roughly 10^3^ times higher and an endurance of 3000 cycles, compared with conventional devices. [47] Cagli [48] et al. reported that a Ti electrode induces the formation of a TiO_x_/HfO_x_ (or TiO_x_N_y_/HfO_x_) interfacial layer in HfO_2_-based RRAM, leaving an oxygen-vacancy-rich Hf-oxide region; this Ti-induced interfacial layer was shown to enable bipolar switching (function iv, interfacial Schottky-barrier modulation, distinct from the bulk work-function effect of function iii). Zhong [49] et al. investigated the effect of the oxygen content of an ITO top electrode on the resistive-switching characteristics of HfO_x_/TiN capacitor structures, reporting that switching parameters such as the SET/RESET voltage and the HRS/LRS resistance values are closely related to the properties of the ITO film; samples with a higher oxygen content in the ITO electrode showed higher resistance in both the HRS and LRS, attributable to the higher resistivity of the ITO film resulting from a lower oxygen-vacancy concentration, and a greater supply of oxygen ions available from the ITO electrode was associated with easier filament rupture during RESET and therefore improved switching stability.

**Bilayer and Doped Architectures for Synaptic Devices.**

Because filamentary-type resistive switching is inherently associated with a broad resistance distribution and non-uniform V_set/V_reset behavior arising from localized filament formation and rupture, bilayer switching-layer structures have been widely explored to stabilize filament formation/rupture, lower switching power, and improve switching uniformity. Ye [50] et al. fabricated a bilayer HfO_2_/TiO_2_ switching layer by magnetron sputtering on a flexible substrate. The switching voltage of the resulting Pt/HfO_2_/TiO_2_/ITO device was lower than that of a Pt/HfO_2_/ITO reference device, because the inserted TiO_2_ layer acts as an oxygen reservoir; the stabilized filament formation/rupture occurring at the HfO_2_/TiO_2_ interface also improved switching uniformity, and analysis of the conduction mechanism showed that the HRS arises from an increase in barrier height with increasing stop voltage. In general, when a bilayer is introduced specifically as an oxygen reservoir or filament-localization layer, three coupled physical quantities are being modified simultaneously, and it is useful to separate them explicitly. First, the local vacancy concentration in the active (filament-forming) layer is reduced at equilibrium because the adjacent reservoir layer offers a lower-energy site for displaced oxygen ions, which raises the effective activation energy the ions must overcome to return and re-form a filament away from the interface—this is what “stabilizes” filament formation/rupture at the bilayer interface rather than allowing it to wander within the active layer. Second, the electric-field distribution across the stack becomes non-uniform: because the reservoir layer and active layer generally differ in permittivity and resistivity, most of the applied voltage drops across whichever layer is momentarily more resistive, so the field seen by the active layer changes dynamically as its own resistance state switches, providing a self-limiting (negative-feedback) effect on filament overgrowth that a single homogeneous layer does not have. Third, the energy landscape for vacancy migration is modified at the internal HfO_2_/TiO_2_ (or analogous) interface itself, where lattice mismatch and differing oxygen affinity create a local potential step that can pin vacancies at the interface; this pinning is the microscopic origin of the improved switching uniformity reported for bilayer devices, including the barrier-height increase with stop voltage noted above. We apply this same three-part framework (vacancy-concentration redistribution, field-division self-limiting, and interfacial pinning) to interpret the remaining bilayer and doped architectures in this subsection.

Ryu and Kim [51] fabricated a Pt/Ti/TiO_2_/Al_2_O_3_/TiN synaptic device incorporating a TiO_x_/Al_2_O_3_ bilayer, demonstrating improved device uniformity together with a gradual increase and decrease in conductance under SET/RESET pulses; interface-type RRAM generally offers good multi-level-cell tunability but slower switching, whereas filamentary RRAM offers fast switching and good endurance at the expense of resistance-state uniformity, motivating the use of oxide bilayers (HfO_2_, TaO_x_, TiO_2_, Al_2_O_3_) to combine the advantages of both.

Yu [52] et al. used hydrothermal synthesis to grow TiO_2_ nanorod arrays and demonstrated that nitrogen doping improves the resistive-switching behavior of magnetron-sputtered TiO_2_ thin-film memristors relative to one-dimensional structures such as nanowires and nanorods that confine the conduction path and thereby improve reproducibility. An Al/N-TiO_2_-nanorod-array/FTO memristor exhibited gradual, bidirectional conductance modulation, good cycling endurance (over 8000 I–V cycles), and an acceptable HRS/LRS ratio, and successfully reproduced key synaptic learning behaviors—paired-pulse facilitation (PPF), short-term memory (STM), long-term memory (LTM), and spike-timing-dependent plasticity (STDP)—required for building artificial-intelligence learning models.

Gul [53] reported a nanoscale, single-layer TiO_2_-based artificial synaptic device, motivated by the observation that a supercomputer solving complex AI problems requires more than 1 MW of power, whereas the human brain consumes roughly 10 W in total; among candidate metal-oxide semiconductors (Hf-, Zn-, V-, Ni-, and Ti-based oxides), TiO_2_ was highlighted as a particularly attractive material because oxygen vacancies act as the primary charge carrier and because of its additional potential in memristors, biosensors, and resistive-switching memory. In this study, the structural characteristics of a 10 nm TiO_2_-based resistive-switching memristor device were confirmed by X-ray photoelectron spectroscopy (XPS) and energy-dispersive X-ray spectroscopy (EDX); depression and potentiation were attributed to oxygen vacancies within the metal-oxide layer, while abrupt resistance changes were attributed to filamentary transitions associated with defects in the active layer.

**Recent Progress in Switching-Layer Materials (2023–2026).**

Materials research on RRAM switching layers has continued to advance rapidly since the studies summarized above. In the HfO_x_ family, Yeh [54] et al. showed that yttrium doping of sputtered HfO_x_ films tunes the oxygen-vacancy formation energy and improves resistive-switching uniformity, while Kim [55] demonstrated that a filamentary HfO_x_-based three-dimensional vertical memristor can achieve highly linear, low-variability six-bit conductance tuning by selectively forming and removing only part of the filament using an incremental-step-pulse-with-verify programming scheme, directly addressing the nonlinearity and dispersion issues discussed above. A self-rectifying HfO_x_/FeO_x_ semiconductor-heterostructure device [56] has similarly been reported to combine a high rectification ratio, a large resistance ratio, and spike-timing-dependent plasticity within a single p–n-junction-like stack. For TaO_x_, Ju [57] et al. showed that inserting a thin SiO_2_ layer into a TaO_x_ switching stack localizes the conductive filament and markedly improves cycle-to-cycle uniformity for neuromorphic applications. Beyond the conventional oxide families, nitride-based switching layers have been reviewed as a route to lower forming voltage and improved thermal stability [58], and multiferroic BiFeO_3_-based resistive switching has been reviewed as an emerging oxide platform combining ferroelectric and resistive-switching functionality [59]. Rare-earth doping strategies, such as Lu-doped HfO_2_ ferroelectric-memristor thin films, have also been explored to realize low-power, multifunctional switching behavior [60]. Two-dimensional (2D) materials have likewise seen substantial recent progress as RRAM/memristor switching and gate-dielectric layers. Zhang [61] et al. reviewed memristors based on 2D hexagonal boron nitride (h-BN), covering synthesis, switching mechanism, and device optimization, while inorganic electrochemical random-access memory (ECRAM) transistors built on bilayer MoS_2_ channels [62] have been shown to combine ionic and electrostatic gating for low-power, highly linear conductance modulation, illustrating the convergence between 2D-material device engineering and analog synaptic-transistor design discussed further in Section 4.

Each material’s score on each axis was assigned by the authors on an ordinal 1–5 scale, ranked relative to the other four materials in this figure only (not against the wider RRAM literature), based on the qualitative statements made about that material in the corresponding “Oxide-Based Switching Layers (OxRAM)” paragraphs of Section 3: for example, TaO_x_ is scored highest on endurance because the “Oxide-Based Switching Layers (OxRAM)” discussion in Section 3 reports it as offering “excellent endurance” and cites endurance figures up to 10\^8 cycles, whereas ZnO and ZrO_2_ are scored lower because no comparably high endurance figure is reported for them in the sources reviewed here. Because the underlying studies differ in device stack, electrode choice, and test conditions, this ranking should be read as a rough, literature-qualitative summary of relative emphasis in the reviewed reports rather than as a benchmarked measurement performed under matched conditions; we are not aware of a single published dataset that measures all five materials side-by-side on all five axes, which is precisely the gap this qualitative figure is intended to summarize rather than substitute for.

## 4. Neuromorphic Functionalities Emulated by RRAM

A biological synapse is the small gap between two neurons through which signals are transmitted; a signal passing from a pre-synaptic to a post-synaptic neuron transiently strengthens the synapse, enabling short-term memory, while repeated stimulation induces longer-lasting changes that support long-term memory. Synaptic devices that emulate this behavior perform learning and memory functions by modulating the weight of an output signal as a function of the intensity, duration, and frequency of the input signal. The principal biological synaptic functions that have been demonstrated in RRAM devices are as follows.

**LTP/LTD (long-term potentiation/depression):** A series of electrical pulses progressively increases (LTP) or decreases (LTD) the device conductance, G, directly corresponding to the weight-update step of an artificial neural network.

**STDP (spike-timing-dependent plasticity):** The magnitude of the synaptic weight change, Δw, is determined by the timing difference, Δt = t_post − t_pre, between pre- and post-synaptic spikes, following the biologically observed learning rule:

$$\Delta w=\left\{\begin{array}{ll} A_{+}\;e^{-\Delta t/\tau_{+}}, & \Delta t>0\\ -A_{-}\;e^{\Delta t/\tau_{-}}, & \Delta t<0 \end{array}\right.$$

Realizing this rule in a two-terminal RRAM device requires that the pre- and post-synaptic timing information be encoded into a single physical voltage waveform seen across the cell, since the device itself has no separate terminals for “pre” and “post” signals. The standard circuit-level approach is to assign each spiking neuron a stereotyped pulse shape—typically a step or exponentially decaying pulse of fixed polarity and amplitude—and to apply the pre-neuron’s pulse to the top electrode and the (time-delayed) complement of the post-neuron’s pulse to the bottom electrode; the net voltage actually appearing across the RRAM cell is then the algebraic difference of the two waveforms, which is engineered so that its integral (or peak overlap) reproduces the desired exponential dependence on Δt. This overlap-based encoding requires, at minimum, a pulse-shaping/waveform generator per neuron, timing/delay circuitry to align pre- and post-spikes at the cell, and a way to prevent the overlap voltage from equaling or exceeding the unwanted SET/RESET threshold outside the intended STDP window; these peripheral-circuit elements add area and power overhead beyond the RRAM cell itself and are a significant part of the ADC/DAC and peripheral-circuit costs referred to in Section 1 and Section 8.

**PPF/PPD (paired-pulse facilitation/depression):** A representative short-term-plasticity (STP) behavior in which the response to a stimulus is modulated by a preceding stimulus applied within a short time interval.

**MLC versus analog synaptic weight storage.** These synaptic functions are sometimes conflated in the RRAM literature with conventional multi-level-cell (MLC) memory operation, but the two target different requirements. MLC operation (as reported for several HfO_x_, TaO_x_, and TiO_x_ devices in the “Oxide-Based Switching Layers (OxRAM)” discussion of Section 3) programs the device into a small number of discrete, well-separated resistance levels (commonly 2–8 levels, i.e., 1–3 bits/cell) using iterative program-verify pulsing, and is optimized for bit-error-rate and retention margin between levels, as in conventional non-volatile storage. Analog synaptic weight storage instead requires a continuous (or very finely graded, often >50–100 distinguishable states) and, ideally, linear and symmetric conductance response to a train of identical pulses, without a verify step, because on-chip learning algorithms update weights incrementally and cannot afford a read–verify–adjust cycle at every step. A device can therefore be an excellent MLC memory (sharp, well-separated levels under program-verify) while being a poor analog synapse (nonlinear, asymmetric, or noisy under open-loop pulsing), or vice versa; where relevant, we indicate below which of the two regimes a given result was obtained in.

Combining the short-term plasticity (STP) and long-term plasticity (LTP) characteristics of RRAM offers a physical route to mitigate catastrophic forgetting and to support lifelong learning, in which important weights are progressively consolidated while less important weights remain dynamically adjustable—analogous to the way the biological brain preserves existing synaptic connections while forming new ones.

**Quantitative synaptic-performance metrics.** Beyond the qualitative LTP/LTD/STDP/PPF behaviors described above, the suitability of an RRAM device for on-chip learning is usually assessed against several more specific figures of merit, which we summarize here because Section 3 and Section 5 refer back to them. (i) Update linearity and symmetry describe how closely the conductance-versus-pulse-number curve follows a straight line, and whether the potentiation (LTP) and depression (LTD) branches are mirror images of one another; strongly nonlinear or asymmetric updates (typical of purely filamentary switching, Section 5) bias gradient-descent-based on-chip training and are usually quantified by a nonlinearity factor extracted by fitting the measured G(pulse) curve to a saturating exponential model. (ii) The number of distinguishable conductance states (sometimes reported as an equivalent bit precision) sets the resolution with which a trained weight can be represented; because this number is degraded by both nonlinearity and read/write noise, it is normally reported as an experimentally distinguishable state count under a defined noise margin rather than as a theoretical maximum. (iii) Dynamic range is the ratio between the maximum and minimum programmable conductance and, together with the number of states, determines the effective weight precision achievable at a given noise floor. (iv) Update (write) noise describes the pulse-to-pulse fluctuation in the conductance change for a nominally identical programming pulse, which is distinct from the device-to-device and cycle-to-cycle variability discussed in Section 5 in that it specifically concerns the analog step size rather than the absolute resistance level. (v) Metaplasticity refers to a change in a synapse’s plasticity rule itself as a function of its prior activity history (for example, a synapse that becomes progressively harder to potentiate the more it has already been potentiated), which is the RRAM analog of the STP-to-LTP consolidation mechanisms noted above and is required for algorithms that seek to protect previously learned weights (Section 1). Individual device reports in Section 3 vary widely in which of these five metrics they report, which limits direct cross-device comparison and is one of the literature gaps this review highlights rather than resolves.

At the array level, the crossbar geometry allows vector–matrix multiplication—the central operation of both artificial and spiking neural networks—to be performed directly in the analog domain by applying Kirchhoff’s and Ohm’s laws to an array of RRAM conductances, as described by equation in Section 1, giving RRAM-based crossbar arrays intrinsic parallelism and energy efficiency for both training-free inference and, potentially, on-chip learning.

Recent reviews have organized these plasticity behaviors around the strategies used to engineer them: Sun et al. [63] categorized plasticity-modulation approaches for neuromorphic devices into chemical, structural, and physical-signal strategies, while three-terminal and multi-terminal memristive architectures have been surveyed as a way to decouple read and write pathways and provide additional, gate-tunable control over plasticity; a representative example is a bifunctional three-terminal, oxygen-ion-migration-based memristor [64] that emulates both synaptic and neuronal (leaky-integrate-and-fire) behavior, including a “learning–forgetting–relearning” cycle, within a single device. Optoelectronic synapses, in which light rather than (or in addition to) an electrical pulse modulates conductance, have also been extensively reviewed [65,66] as a complementary route to STDP- and PPF-like plasticity with reduced RC delay and parasitic heating.

## 5. Non-Ideal Characteristics and Reliability Challenges

Realizing accurate on-chip learning in neuromorphic hardware requires the device conductance to change linearly and symmetrically with pulse number. In practice, however, RRAM devices exhibit several categories of non-ideal behavior that limit both training accuracy and array-level reliability.

**On-chip training versus inference-only accelerators.** The severity of every non-ideality discussed in this section depends strongly on whether the target application is on-chip (in situ) training or inference-only acceleration of weights trained off-chip. Inference-only crossbars program each cell once (or infrequently, for periodic refresh) and are therefore comparatively tolerant of update nonlinearity/asymmetry and write noise, since these only matter at programming time; their dominant requirements are read-disturb immunity, retention, and read-level separation. On-chip training, in contrast, repeatedly updates every cell over the course of training and is therefore highly sensitive to update linearity/symmetry, write noise, and endurance, in addition to retention. We flag which regime is most relevant as each non-ideality is discussed below.

**Nonlinearity and asymmetry.** Because most RRAM devices switch through the abrupt formation and rupture of a conductive filament, their LTP (potentiation) and LTD (depression) conductance-update curves are typically strongly nonlinear and asymmetric. This nonlinear weight-update behavior significantly amplifies the backpropagation error when neural-network training algorithms are applied, sharply reducing hardware inference/training accuracy relative to software simulation—an effect that matters primarily for on-chip training, as noted above, since a fixed inference weight is unaffected by the shape of the update curve used to program it.

**Device-to-device variability.** RRAM switching is a stochastic process governed by the random diffusion and recombination of oxygen vacancies (or metal ions). Device-to-device (D2D) variation causes the initial resistance, threshold voltage, and conductance range to differ across nominally identical devices on the same chip, with a physical origin in the random nucleation site and local defect density that governs where the first conductive filament forms during electroforming; because this is set once, at fabrication and forming, D2D variability is often reported as a static, per-die or per-wafer distribution (e.g., a coefficient of variation on HRS/LRS resistance) that must be calibrated out at the system level (for example, per-cell reference or offset correction) rather than corrected by further electrical cycling. D2D variability is the dominant error source for inference-only accelerators, since it directly sets the achievable read-margin between adjacent weight levels.

**Cycle-to-cycle variability.** Cycle-to-cycle (C2C) variation causes the conductance change to vary from pulse to pulse even within a single device, with a physical origin in the stochastic, atom-by-atom nature of filament formation/rupture at each individual SET/RESET event, superimposed on a given device’s fixed D2D characteristics; because it re-randomizes on every switching event, C2C variability cannot be calibrated out with a one-time correction and instead accumulates as noise over repeated on-chip weight updates, making it the dominant error source for on-chip training rather than for inference. Reported C2C figures (typically expressed as the pulse-to-pulse standard deviation of the programmed resistance level, normalized to the mean) are generally comparable in magnitude to D2D figures for the same device but are, by the argument above, not interchangeable with them, and studies that quote only a single combined “variability” number without separating the two conflate a static, correctable error with a dynamic, accumulating one. Random telegraph noise (RTN), arising from fluctuating trap states near the filament, further degrades the signal-to-noise ratio and contributes to C2C variability specifically, since it is a time-dependent fluctuation superimposed on an otherwise stable programmed state.

**Sneak-path current and the 1S1R trade-off.** In a crossbar array, unwanted current can flow through unselected cells via parallel sneak paths, causing read/write errors and a sharp increase in power consumption. Suppressing this effect requires a select device (selector) in series with each RRAM cell—a 1S1R (one-selector–one-resistor) architecture. Because the selector itself must simultaneously provide an excellent on/off ratio and highly uniform switching, integrating 1S1R arrays substantially increases process complexity, and this complexity is not merely a fabrication inconvenience but translates into a concrete set of system-level trade-offs: the selector occupies additional cell area (a 1S1R cell is typically larger than a bare 1R cell, partly offsetting the density advantage of the “3D Vertical RRAM (VRRAM) Patterning” approach in Section 6), it adds a series voltage drop that increases the total SET/RESET voltage the peripheral driver circuit must supply, its own current-voltage nonlinearity and threshold-voltage variability propagate into the effective read margin of the RRAM cell in series with it, and its deposition/anneal budget must be compatible with the RRAM stack’s own thermal budget (the “BEOL CMOS-Compatible Integration” discussion in Section 6), which constrains the choice of selector material as much as its raw switching performance does. These area, voltage, variability, and fabrication-complexity penalties should therefore be weighed against the sneak-path suppression a selector provides, rather than treating 1S1R integration as a cost-free solution. Recent work continues to push both sides of this trade-off: Seo and Yang [67] improved the cycling reliability of a GeSeTe ovonic-threshold-switching (OTS) selector integrated with a Pt/LiNbOx/W RRAM cell in a 4-kilobit 1S1R crossbar array by combining DC-sweep SET with single-pulse AC RESET, achieving endurance beyond 10^12^ cycles with sub-100 ns switching and device-to-device variability below 5%, while Chen et al. [68] showed that boron doping of a GeSe-based OTS selector can reproduce arsenic-like nonlinearity and thermal stability without using toxic arsenic, illustrating how selector-material engineering remains an active complement to the RRAM-materials strategies discussed in Section 3.

**IR-drop.** As array size increases, voltage drop along the interconnect resistance (IR-drop) becomes more severe, so that devices at the periphery of a large array receive a lower effective voltage than intended, degrading weight-update accuracy and overall computational precision.

Recent device-level studies continue to target these variability and nonlinearity issues directly: nickel-doped and tin-doped ZnO-based memristors have been reported [69,70] to improve threshold-switching and conductance-modulation linearity for neuro-inspired computing, illustrating that dopant and composition engineering, similar to the HfO_x_ and TaO_x_ strategies described in the “Recent Progress in Switching-Layer Materials” part of Section 3, remains an active route to mitigating device-to-device and cycle-to-cycle variability.

**Endurance.** On-chip training requires a large number of repeated weight updates (potentially in the billions over a full training run for a large network, though the number scales with dataset size, batch size, and epoch count and is workload-dependent rather than fixed), but repeated switching cycles progressively and permanently degrade the insulating layer, limiting the achievable number of switching cycles to roughly10^6^–10^8^ for a bare RRAM cell under continuous SET/RESET cycling. This nominal endurance window can be substantially short of what a full on-chip training run demands for a large model trained over many epochs, which has motivated several endurance-extending strategies at the array and algorithm level rather than the device level alone: weight-transfer schemes that periodically move a frequently updated analog weight to a more stable digital or off-chip representation once it has converged, redundancy schemes that map a single logical weight across multiple physical cells and average or vote across them to tolerate individual-cell wear-out, refresh schemes that periodically rewrite a cell to its nominal state to reset accumulated drift before it approaches a failure threshold, and write-reduction (sparse-update) training algorithms that skip a weight update for cells whose gradient contribution is below a threshold, directly reducing the number of switching cycles a given cell must sustain over training. For inference-only accelerators, by contrast, endurance is rarely the limiting factor because cells are programmed once or refreshed only occasionally.

**Retention.** Retention is likewise imperfect: although RRAM is nominally non-volatile, thermally activated diffusion of oxygen vacancies at elevated temperature can cause the stored conductance state to drift or be lost over time, and this is not well captured by a single retention-time figure at one temperature and one resistance level. Reported retention times generally decrease with increasing ambient or self-heating temperature, following the same thermally activated (Arrhenius-type) kinetics that govern the switching models described in Section 2; they also typically depend on the programmed resistance level itself, since a lower-resistance (larger/denser filament) state is usually more thermally stable than a high-resistance or intermediate analog state, making multi-level and analog states generally more retention-limited than the binary LRS/HRS extremes; retention further depends on the specific filament configuration produced by a given SET history (e.g., filament diameter and vacancy density), so nominally identical programmed conductance values reached via different pulse histories are not guaranteed to drift identically; and retention degrades further with accumulated switching cycles as the device approaches its endurance limit, i.e., a device’s retention and endurance characteristics are coupled rather than independent specifications. For these reasons, we treat published retention figures in Section 3 as illustrative of a given stack under its reported test condition rather than as directly comparable, single-number device specifications.

## 6. Process Technology for RRAM Integration

Fabricating RRAM devices over a large area with high yield and integrating them at high density on production-scale semiconductor lines requires continued advances in the following process technologies.

Because RRAM switching characteristics are extremely sensitive to the quality of the few-nanometer-thick insulating layer, ALD—based on self-limiting surface reactions—is used to deposit HfO_2_, Al_2_O_3_, and related films with excellent atomic-scale thickness uniformity. When forming an oxygen-deficient conductive layer such as HfO_x_, the oxygen concentration (the value of x) can be precisely tuned by adjusting the ALD cycle ratio, providing control over the device’s initial resistance state and set voltage.

The condition of the interface between the electrode and the insulating layer strongly influences current behavior. Inserting an oxygen-reservoir layer—a thin TiN or metal film—between the active electrode (e.g., Ti, Ta) and the switching layer allows the process to temporarily store oxygen ions released during the SET operation and release them again during RESET, an interface-engineering strategy that is essential for reliable switching. Post-deposition plasma treatment (oxygen or nitrogen plasma) is also used to passivate unwanted surface traps and defects, suppressing leakage current.

One of the principal advantages of RRAM is that it can be integrated directly on top of a pre-fabricated silicon CMOS logic circuit. To avoid thermally damaging the underlying logic transistors, RRAM fabrication is typically integrated into a low-temperature (below ~400 °C) back-end-of-line (BEOL) metallization flow. In a 1T1R architecture, one RRAM cell is stacked vertically on one transistor, which is intended to suppress sneak-path current within that cell; achieving this in practice also requires the transistor and RRAM element to be well matched in leakage and breakdown voltage, and the 1T1R approach trades this benefit for a larger cell area (typically several F^2^ larger than a bare 1R cell) and forms the basis of a neuromorphic core.

To overcome the area limitations of planar (2D) crossbar arrays, three-dimensional stacking approaches analogous to 3D NAND flash are being introduced. In a typical process, metal electrodes and insulating films are alternately deposited in tens of layers, a single high-aspect-ratio anisotropic dry-etch step opens a deep hole through the stack, and the switching material (e.g., HfO_2_) is then conformally coated onto the sidewalls by ALD. Because increasing the number of layers does not proportionally increase the number of lithography steps, this approach dramatically increases synaptic-array density while limiting the increase in process cost.

The reliability and variability challenges facing neuromorphic hardware are being addressed through combined materials and process design rather than through a single approach alone: Nanotexturing the bottom-electrode surface into nanopyramid or tip geometries concentrates the electric field at a specific location, pinning the otherwise randomly formed filament position and substantially improving both D2D and C2C variability. The localized Joule heating generated during filament formation/rupture degrades device endurance; high-thermal-conductivity electrode materials or capping-layer processes are used to rapidly dissipate heat from the switching region and suppress degradation—this switching-level, nanosecond-scale, filament-localized Joule heating is a distinct phenomenon from the chip-level heat accumulation discussed in the “Thermal Management for Densely Integrated RRAM” part of Section 7, which develops over millisecond-to-second timescales as many thousands to millions of cells switch across a die. The two require different mitigation strategies (local thermal design of the cell stack versus package-level heat spreading) and are treated separately in this review. Depositing a high-performance selector material (e.g., an Ovonic threshold switch, OTS) in series with the RRAM layer is process-intensive because differing deposition temperatures and interdiffusion between materials must be avoided; inserting atomic-scale diffusion-barrier layers to solve this problem is expected to be a key materials/process research topic for future volume production of neuromorphic chips.

## 7. Neuromorphic Packaging

**Figure 4:**
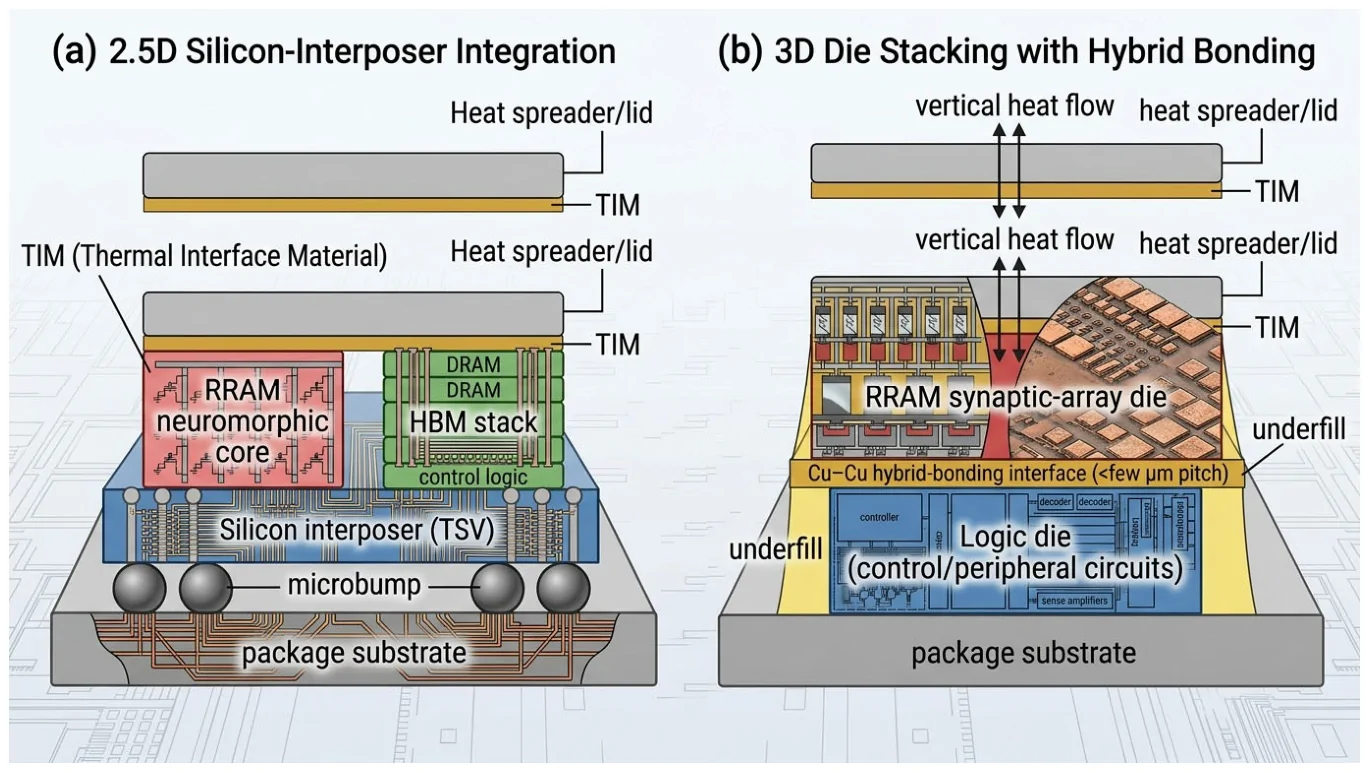
Schematic cross-sections of 2.5D silicon-interposer and 3D hybrid-bonding packaging architectures for RRAM-based neuromorphic chips. Schematic cross-sections of two heterogeneous-integration strategies: (**a**) 2.5D integration, in which an RRAM neuromorphic core and HBM stack are placed side by side on a silicon interposer with through-silicon vias (TSVs) and microbumps; and (**b**) 3D die stacking, in which an RRAM synaptic-array die is bonded directly onto a logic die through a Cu–Cu hybrid-bonding interface, with underfill and a thermal-interface material (TIM)/heat-spreader stack for mechanical and thermal management [71].

For RRAM-based neuromorphic systems to move beyond the single-device or unit-array level to commercially viable AI accelerators and on-device AI hardware, advanced semiconductor packaging is essential. Although RRAM-based neuromorphic chips offer extremely high parallel-computation efficiency, their high integration density brings substantial packaging challenges: rising power density, the need for data communication with heterogeneous chips, and the requirement for structural reliability (Figure 4).

**2.5D/3D Heterogeneous Integration and Chiplets.**

Modern AI computation is rarely performed by an RRAM-based, in-memory-computing (PIM) core alone; a complete system typically also requires a control-logic processor, a high-speed data I/O interface, and separate high-performance memory (e.g., HBM) for bulk data storage. 2.5D silicon-interposer integration places the neuromorphic core and high-bandwidth memory (HBM) side by side on a silicon interposer using fine-pitch microbumps to maximize bandwidth; as next-generation memories such as HBM4 are integrated with neuromorphic compute chips, maintaining signal integrity through the interposer becomes a central packaging challenge.

3D die stacking and hybrid bonding use through-silicon vias (TSVs) to vertically stack a die containing the RRAM array with a logic die. Hybrid bonding, which directly joins copper pads without microbumps, is increasingly used to shrink the inter-chip interconnect pitch to the few-micrometer scale and to eliminate energy loss from parasitic capacitance. As a recent example, die-to-wafer and wafer-to-wafer hybrid-bonding processes with a pitch below 2.5 µm have been demonstrated for 3D chiplet AI applications [71], reducing the thermal resistance per interconnect layer by roughly an order of magnitude relative to conventional solder-based bonding—directly relevant to the thermal-management challenge discussed.

Chiplet architectures subdivide a large neuromorphic chip into smaller functional chiplets, which are then reassembled using advanced packaging (system-in-package, SiP) techniques to address yield limitations, an approach that has become essential in the neuromorphic ecosystem as well. Thermal management issues for densely integrated RRAM are also a very important issue in packaging.

**Thermal Management for Densely Integrated RRAM.**

RRAM devices inevitably generate localized Joule heating during the formation and rupture of the conductive filament. When billions of synaptic devices switch simultaneously in a densely integrated 2.5D/3D neuromorphic chip, severe local heat accumulation can occur, degrading device retention and causing system malfunction. Thermal-filler-based advanced thermal-interface materials (TIMs) are required to rapidly conduct heat away from the die to a heat spreader; packaging-materials research increasingly focuses on dispersing high-efficiency thermal fillers—alumina (Al_2_O_3_), aluminum nitride (AlN), and boron nitride (BN)—at high loading within a polymer matrix while forming an optimal percolation network. Direct cooling and heat-spreading structures address the thermal-trap phenomenon in which heat generated at a lower die in a 3D stack cannot easily escape upward; physical solutions include etching microchannels within the silicon substrate for liquid cooling and densely placing dummy thermal TSVs to forcibly increase vertical thermal conductivity. In 3D-stacked and 2.5D interposer structures, the mismatch in coefficient of thermal expansion (CTE) between the chip and the substrate imposes severe mechanical stress on solder joints and microbumps. As bump pitch continues to shrink, the underfill process protecting these interconnects faces new limits.

**Next-Generation Underfill and Nanocomposite Processes.**

High-flow, fine-pitch underfill technology is required so that capillary underfill (CUF), non-conductive film (NCF), and non-conductive paste (NCP) can completely fill the narrow gap between neuromorphic dies at HBM4-level ultra-fine pitch without void formation. Electrospinning and electrospray processes are being introduced into packaging materials to form uniform, nanoscale thermal and reinforcing networks beyond what conventional mixing or film-casting processes can achieve. Electrospinning, in which a high voltage is applied to a polymer solution to draw out nanofiber mats, can create underfill composite films in which thermal fillers are highly aligned within one-dimensional nanochannels embedded in the epoxy matrix. Electrospray can likewise be used to coat insulating, thermally conductive nanoparticles as an ultrathin, uniform layer on the packaging substrate or device surface, minimizing the thermal resistance between chip and interposer and maximizing interfacial adhesion—research directions expected to substantially improve the reliability of neuromorphic packaging. **Scope note on RRAM-specificity.** We should be explicit that electrospinning/electrospray underfill processing, and much of the thermal-interface-material and 2.5D/3D integration discussion in this section more broadly, has so far been demonstrated and reported primarily as general advanced-packaging technology for high-density logic and HBM-class memory stacks, not as a solution developed for, or specifically validated on, RRAM neuromorphic arrays. We include it here because the packaging requirements it targets—fine-pitch interconnect, CTE-mismatch stress, and thermal-interface resistance—are shared by any densely stacked die, RRAM-based or not, and because no RRAM-specific underfill or packaging literature comparable in maturity currently exists to our knowledge. Where a technology in this section has, to our knowledge, actually been demonstrated on an RRAM or memristor neuromorphic die (as opposed to being a general packaging concept we judge to be applicable), we have indicated so explicitly (e.g., the hybrid-bonding pitch data in the “2.5D/3D Heterogeneous Integration and Chiplets” discussion of Section 7); the remainder, including this subsection, should be read as a projection of general advanced-packaging capability onto RRAM neuromorphic requirements rather than as a demonstrated RRAM-specific result, and is offered as a research direction rather than an established solution.

**Summary and Future Direction for Neuromorphic Packaging.**

The successful commercialization of RRAM-based neuromorphic devices cannot be achieved through device-level excellence alone; it requires co-optimization with packaging technologies that combine thermal management, stress control, and ultra-high-speed interconnects. Three priorities stand out: (i) standardizing 3D/chiplet form factors, including standard interfaces (e.g., UCIe) that seamlessly connect heterogeneous RRAM compute tiles, control logic, and HBM through hybrid bonding and silicon-interposer-based chiplets; (ii) materials innovation in thermal management, combining optimized thermal-filler networks with advanced cooling structures to control hotspots arising from dense neuromorphic cores; and (iii) advanced nanoprocess-integrated underfills, actively adopting new nanoscale processes such as electrospinning to develop next-generation underfill composites capable of ensuring thermomechanical reliability even at ultra-fine pitch. Future neuromorphic packaging will therefore serve not merely to protect devices and provide electrical connections, but as a system-level enabler that directly determines a chip’s computational performance, lifetime, and power efficiency.

## 8. Applications and Comparison with Competing Non-Volatile Memory Technologies

The term “neuromorphic computing” was coined by Carver Mead in 1990 to describe a computing paradigm inspired by the cognitive function of the human brain. In today’s data-centric world, where extracting meaningful information from large volumes of unstructured data is one of the most valuable computing tasks, neuromorphic computing offers low-power, high-performance computation. Realizing the multiply–accumulate (MAC) operations that occur between neurons with higher throughput and energy efficiency, however, requires specifically designed hardware (integrated circuits and devices) rather than software emulation alone.

Because neuromorphic-computing architectures are specifically designed for higher energy efficiency and greater parallelism in big-data processing, non-volatile-memory-based synapses offer superior device scalability and array density and are more energy efficient than volatile-memory-based synapse arrays. Hardware artificial-neural-network (ANN) systems built from high-density synaptic-array devices can perform large-scale parallel computation for pattern recognition at low power. Realizing a neuromorphic system with on-chip training capability, however, requires an ideal synaptic device that simultaneously satisfies a wide range of requirements: scalability, MLC behavior, low-power operation, data retention, and symmetric/linear conductance change in both the potentiation and depression modes [72,73,74,75]. RRAM technology is generally described as compatible with existing CMOS processes, though this claim should be substantiated rather than asserted on the basis of low-temperature BEOL integration alone (the “BEOL CMOS-Compatible Integration” discussion in Section 6). Manufacturing-level CMOS compatibility in practice depends on: thermal budget, i.e., whether every deposition and anneal step in the RRAM stack stays within the ~400 °C ceiling set by underlying back-end-of-line metallization and any already-formed transistors; contamination control, since several active-metal and dopant species used in RRAM switching layers and electrodes (e.g., Cu, Ag) are known contaminants in standard front-end-of-line CMOS fabs and require dedicated tooling or back-end-only integration to avoid cross-contamination; electrode integration, including adhesion, diffusion barriers, and etch selectivity when patterning the RRAM stack against existing interconnect metals; lithography requirements, which are relatively relaxed for a single-layer 1R array but become a limiting factor for 1T1R and 3D VRRAM integration (“3D Vertical RRAM (VRRAM) Patterning,” Section 6), where alignment tolerance across many stacked layers must be maintained; wafer-scale yield and uniformity of the forming voltage and initial resistance distribution across a full wafer, which is a separate question from single-device switching demonstrations and is rarely reported alongside them; and BEOL reliability under subsequent thermal cycling from packaging (Section 7) and long-term operation. Individual demonstrations reviewed in Section 3 typically address only a subset of these requirements (most often thermal budget and basic electrode integration), so “CMOS-compatible” as used in this review and in Table 1 should be read as “compatible with at least the low-temperature BEOL integration route,” not as a claim that full manufacturing-line qualification has been demonstrated. RRAM-embedded systems can be integrated into IoT, automotive, and infotainment platforms. Beyond the memory hierarchy, RRAM can implement neuromorphic computing both as a non-volatile logic circuit element and as a synaptic device for low-power computing. Given the currently limited synaptic characteristics of two-terminal RRAM devices, bit-precision-limited hardware ANNs that rely on off-chip training remain the most practical near-term configuration. RRAM-based synaptic devices have been studied extensively over the past decade, but a stable, reliable device that fully satisfies every synaptic-device requirement has not yet been developed; because the conductance change of an RRAM device is directly linked to the atomic-scale migration of oxygen vacancies or metal ions under an electric field, simultaneously minimizing variability and maximizing conductance remains difficult. In addition, symmetric and linear conductance change under constant training pulses is a key requirement for improving pattern-recognition accuracy. As one example of ongoing work in this area, Wu [76] et al. proposed a new electro-thermal methodology to improve the linearity of filamentary analog RRAM and demonstrated devices with excellent linearity for both SET and RESET operations.

RRAM also competes and coexists with several other emerging non-volatile memory technologies being developed for neuromorphic hardware. Table 1 summarizes the key characteristics of RRAM (resistive switching), PCRAM (phase-change), STT-MRAM (spin-transfer-torque magnetic), FeRAM/FTJ (ferroelectric), and ECRAM (electrochemical, ionic).

**Energy efficiency as a comparison metric.** Because the central motivation for neuromorphic computing given in Section 1 is energy-efficient computation rather than raw switching speed or density, Table 1 is incomplete without an explicit energy comparison, and device switching energy alone is not sufficient to evaluate a candidate technology as an AI accelerator. A more complete comparison distinguishes at least four energy quantities that are frequently conflated in the device literature: (i) energy per programming (write) event, i.e., the energy dissipated in a single SET or RESET pulse at the device level, which is what is usually reported in the materials studies cited in Section 3 (commonly ranging from sub-pJ to several nJ per event across the RRAM literature, depending strongly on compliance current and pulse width, versus tens of pJ for PCRAM RESET and sub-pJ regimes reported for some STT-MRAM and ECRAM cells); (ii) energy per MAC (multiply–accumulate) operation at the array level, which includes the analog read energy of every cell contributing to a dot product and is the more relevant figure for inference workloads; (iii) read energy, generally much lower than write/programming energy for all the technologies in Table 1 but non-negligible when a crossbar is read at high frequency during inference; and (iv) system-level energy efficiency, typically reported in TOPS/W (tera-operations per second per watt) or pJ/MAC once ADC/DAC conversion, peripheral CMOS, and interconnect energy are included—the quantities we noted in Section 1 as absent from a purely device-level O(1)-complexity argument. Because published RRAM neuromorphic demonstrations report these four quantities inconsistently and often report only (i), we do not extend Table 1 with a numerical energy row; a fair, consistent TOPS/W-level comparison across RRAM, PCRAM, STT-MRAM, FeRAM/FTJ, and ECRAM prototypes reported under comparable array sizes and precision would require a dedicated survey and is, in our assessment, itself a gap in the current review literature.

Both competing technologies summarized in Table 1 continue to advance rapidly. On the phase-change side, Syed et al. [77] reviewed the chemistry and device engineering of phase-change memory for in-memory computing, highlighting continued progress in multilevel-cell programming and drift mitigation that keeps PCRAM competitive with RRAM for large-scale analog accelerators. On the magnetic side, spin-orbit-torque MRAM (SOT-MRAM) has been reviewed as a route to faster switching and higher endurance than conventional STT-MRAM, though field-free switching and back-end-of-line integration remain open challenges [78]; at the device level, a domain-wall magnetic-tunnel-junction structure has recently been demonstrated to implement both artificial synapses and neurons within an all-spin neuromorphic hardware platform, illustrating that MRAM-based technologies are pursuing the same synapse-and-neuron co-integration goal discussed for RRAM in Section 2 and Section 4 [79].

## 9. Future Outlook

**Figure 5:**
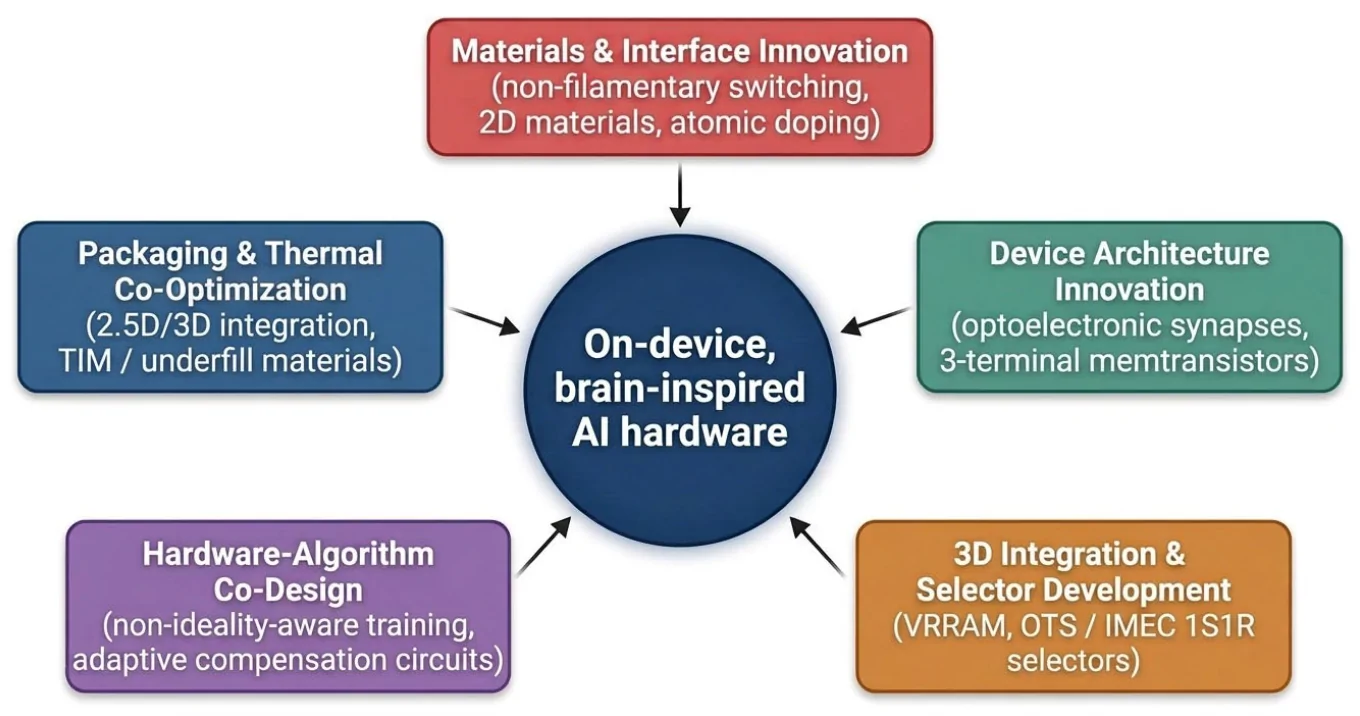
Convergent research directions for future RRAM-based neuromorphic hardware. Convergent research directions for future RRAM-based neuromorphic hardware, corresponding to the topics discussed in Section 9: materials and interface innovation, device-architecture innovation, 3D integration and selector development, and hardware–algorithm co-design, together with the packaging and thermal co-optimization discussed in Section 7. These five directions are pursued in parallel and converge toward on-device, brain-inspired AI hardware [71,80,81].

Throughout this section, we distinguish, wherever possible, technology that has already been experimentally demonstrated at the single-device or small-array level (which we describe using demonstrated, reported, or achieved) from technology that remains at the level of a proposed direction, simulation, or extrapolation from related fields (which we describe using is expected to, could enable, or a research direction). The “3D Integration and Selector Development” discussion’s 3D VRRAM patterning and the “Hardware–Algorithm Co-Design” discussion’s open-source training toolkits, for example, are demonstrated and cited to specific reports; the femtojoule-level switching energy mentioned in the “Materials and Interface Innovation” discussion and the fully autonomous on-device learning vision of the “Toward Autonomous On-Device Learning” discussion, by contrast, are extrapolations that have not, to our knowledge, been jointly demonstrated in a single RRAM neuromorphic system and should be read as motivating goals rather than established results (Figure 5).

Because random, filamentary switching is an intrinsic source of stochastic variability, analog resistive-switching materials that instead rely on a uniform, interfacial barrier-modulation mechanism—such as PrCaMnO- or SrTiO_3_-based systems, or doped HfO_2_-family compounds—are being developed at an accelerated pace as non-filamentary interface-switching devices. Two-dimensional materials and atomic-layer thin-film processes are also attracting substantial interest: stacking two-dimensional semiconductors such as graphene, hexagonal boron nitride (h-BN), and transition-metal dichalcogenides (TMDs) allows the atomic-scale filament growth pathway to be controlled directly, dramatically reducing switching variability and lowering power consumption toward the femtojoule (fJ) per switching-event level. Atomic-scale doping and multilayer optimization—for example, trace doping with Al, Ti, or N to tune the activation energy for oxygen-ion migration, combined with layered structures such as HfO_x_/TaO_x_ or Al_2_O_3_/HfO_x_—are similarly being used to homogenize the residual electric-field distribution and secure switching linearity.

Optoelectronic synaptic devices, in which light (photons) rather than an electrical stimulus serves as the signal carrier, are emerging as a way to avoid the RC delay and heat generation associated with parasitic capacitance, maximizing bandwidth and enabling ultra-fast, ultra-low-power synaptic operation. Three-terminal, gate-controlled memristive devices (memtransistors) are also gaining attention as a way to resolve the read-disturb problem inherent to conventional two-terminal devices, by adding a gate electrode that separately controls conductance and thereby dramatically improving linearity and symmetry.

Vertical RRAM (3D VRRAM), fabricated using plug processes analogous to 3D NAND flash, continues to be pursued as a core technology for manufacturing next-generation, ultra-high-density in-memory-computing chips. In parallel, self-rectifying and threshold-switching selectors—based on Ovonic threshold switch materials or mixed ionic–electronic conduction (MIEC) systems—remain essential for eliminating leakage current while preserving a small cell footprint and are being pursued alongside high-density 1S1R integration technology.

Because it is extremely difficult, from a purely physical standpoint, to fully eliminate the non-ideal characteristics of an individual device, circuit- and software-level compensation techniques are being developed in parallel. Non-ideality-aware training pre-incorporates measured device nonlinearity, noise, and D2D variability into the training simulation before weights are mapped onto hardware. On-chip variability-compensation circuits—for example, adaptive input-pulse schemes that calculate IR-drop within the array in real time and adjust the applied pulse width or voltage accordingly—are similarly improving computational precision. Rasch et al. [80] developed a hardware-aware retraining methodology that systematically evaluates analog in-memory computing accuracy across diverse deep-learning topologies (convolutional, recurrent, and transformer networks) by explicitly modeling crossbar-level non-idealities, showing that many large-scale networks can be retrained to reach iso-accuracy with their floating-point counterparts. To make such co-design methodologies broadly accessible, Le Gallo et al. [81] released an open-source analog in-memory hardware acceleration kit that couples hardware-calibrated device models with standard deep-learning training pipelines, allowing device engineers and algorithm designers to jointly evaluate how RRAM-array non-idealities—of exactly the kind summarized in Section 5—propagate into end-to-end network accuracy before committing to silicon.

Today’s AI systems remain heavily dependent on cloud infrastructure because of their large computational and power requirements. Once RRAM-based synaptic arrays mature, however, real-time, on-device data processing at milliwatt-level power should become possible, opening the door to fully on-chip training in which edge devices continually fine-tune their weights in response to a changing environment. Future neuromorphic hardware is expected to move beyond planar 2D crossbar arrays toward 3D vertically stacked (VRRAM) architectures that exponentially increase integration density, and hardware is expected to evolve from a passive executor of software into a full partner with brain-inspired AI algorithms that directly exploit the physical switching characteristics of RRAM—dynamic plasticity, stochastic variability, and so on—as computational primitives in their own right.

## 10. Conclusions

RRAM-based neuromorphic computing represents one of the most promising technology candidates for realizing ultra-low-power, highly parallel, and densely integrated AI hardware beyond the limits of the von Neumann architecture. While RRAM devices boast excellent CMOS process compatibility, rapid switching speed, and outstanding scalability, significant challenges hinder their field deployment. These include nonlinear and asymmetric conductance modulation, device-to-device variability driven by stochastic ion migration, and parasitic issues such as sneak-path currents and IR-drop within crossbar arrays. To overcome these limitations, future developments in neuromorphic RRAM technology must focus on three distinct levels: Maximizing linearity and uniformity by developing novel interface-controlled switching materials and hybrid two-dimensional (2D)/thin-film heterostructures at the single-device level. Constructing ultra-high-density 3D vertical RRAM (VRRAM) architecture while ensuring stable integration with highly selective 1S1R selector technologies. Establishing a hardware–software co-design framework in which intrinsic device non-idealities are actively absorbed and compensated for at both the circuit and algorithmic levels at the system level. If this multi-pronged, convergent research program succeeds, RRAM-based neuromorphic devices are expected to play a central role in the future of computing, including on-device AI, autonomous driving, robotics, and large-scale artificial-neural-network accelerators. RRAM currently combines a simple device structure with a wide range of organic and inorganic materials for the insulating layer and electrodes, and continues to be improved through multi-level-cell (MLC) operation, multi-step forming techniques, transparent RRAM devices based on ZnO, vertical low-power ReRAM implementations, and a variety of thin-film deposition techniques—including PLD, PEALD, and sputtering. Together with its non-volatility and CMOS compatibility, these advances continue to support the case for RRAM as a next-generation, synapse-based neuromorphic memory device; nevertheless, because a stable RRAM device satisfying every synaptic-device requirement, including conductance-tuning linearity, has not yet been fully realized, continued research in this area remains necessary.

## Figures and Tables

**Table 1:** Comparison of RRAM with competing non-volatile memory technologies for neuromorphic computing.

Parameter	RRAM (Resistive)	PCRAM (Phase-Change)	STT-MRAM (Magnetic)	FeRAM/FTJ (Ferroelectric)	ECRAM (Electrochemical)
Operating principle	Conductive filament via oxygen-vacancy/metal-ion migration	Crystalline/amorphous phase change	Spin-polarization-dependent tunnel magnetoresistance	Change in ferroelectric polarization state	Resistance change via ion doping
Linearity/symmetry	Moderate to low (improving)	Low (abrupt RESET)	Low (strongly binary)	Good	Excellent (analog)
Switching speed	<10 ns	~50 ns	<10 ns	<10 ns	~µs (slow)
Endurance (cycles)	10^6^–10^8^	10^8^	>10^12^	10^10^–10^12^	>10^9^
Scalability	Excellent (<10 nm)	Good	Moderate	Moderate to good	Limited (three-terminal structure)
Process compatibility	Good (CMOS-compatible)	Good	Complex	Good (HfO_2_-based)	Limited (new materials/electrolyte)

## Data Availability

No new data were created or analyzed in this study. Data sharing is not applicable to this article.
